# *‘People don’t realise how much their past experiences affect them in adulthood’*: A qualitative study of adult siblings’ experiences of growing-up with a sister/brother with a childhood life-limiting condition and their perceived support needs

**DOI:** 10.1177/02692163231225100

**Published:** 2024-02-11

**Authors:** Susan Kirk, Steven Pryjmachuk

**Affiliations:** School of Health Sciences, University of Manchester, Manchester, UK

**Keywords:** Siblings, child, palliative care, hospices, qualitative research

## Abstract

**Background:**

There is a lack of research about the experiences and impact of having a sibling with a life-limiting condition. Studies focus on the sibling experience during childhood but the experience and impact during adulthood is unknown despite the increased life-expectancy of children with life-limiting conditions.

**Aim:**

To explore adult siblings’ perspectives on the experience of having a sister/brother with a childhood life-limiting condition and to identify their perceived needs and preferences for support.

**Design:**

A qualitative exploratory study design with data generated by semi-structured interviews and analysed using reflexive thematic analysis, underpinned by interpretivism.

**Setting/participants:**

Twenty-two siblings (17–42 years old) were recruited via a children’s hospice in England.

**Results:**

The experience of having a sibling with a life-limiting condition changes over time in response to how understandings of the meaning of a life-limiting condition develop and changing roles/relationships with parents and siblings. These experiences have an enduring impact on adult sibling’s mental health which is compounded by their unmet (and sometimes unrecognised) support needs in adolescence and adulthood. Siblings described the importance of support continuing into adulthood with a focus on the provision of psychotherapy and peer support.

**Conclusions:**

Having a sister/brother with a childhood life-limiting condition appeared to have a significant and ongoing impact on adult siblings but their support needs, particularly for psychotherapy and peer support, are overlooked. The findings highlight the importance of ensuring siblings are included in family assessments and that family-based interventions are developed to promote sibling-parent relationships.

**What is already known about this topic?**

- Research suggests that during childhood the siblings of children with a life-limiting condition can experience emotional and behavioural difficulties and have a lower quality of life.
- There has been a lack of research about adult siblings’ experiences and support needs despite the increasing prevalence and life expectancy of children and young people with life-limiting conditions.

**What this paper adds?**

- Bereaved and non-bereaved adult siblings experience mental health problems that have endured from childhood/adolescence.
- Siblings’ needs can be overlooked by parents during childhood/adolescence which influences their relationships in adulthood.
- Siblings report unmet needs for psychotherapy and peer support during adolescence and adulthood.

**Implications for practice, theory or policy**

- Palliative care services should provide appropriate psychotherapy and peer support for the adult siblings of children and young people with life-limiting conditions.
- Palliative care services need to promote open parent-sibling communications and include siblings in holistic family assessments to ensure their individual support needs are identified.
- Greater priority should be given to support for siblings across the life course in palliative care policy and practice.

## Background

The sibling relationship is generally considered to be the longest lasting human relationship and one that influences an individual’s emotional, social and cognitive development.^1,2^ It is normatively characterised as being egalitarian, reciprocal and mutual with siblings seen as a source of support and companionship.^3–5^ In addition, the relationship is presented as changing over the life course and to be influenced by a range of personal, interpersonal and environmental variables.^5,6^ One significant influence on the relationship and overall sibling experience is that of having a disabled sibling or one with a long-term or life-limiting condition. Siblings who have a sister/brother with a life-limiting condition are likely to experience their early death which may have profound and life-long implications that disrupt their own life course trajectory.

While research has examined the lives of siblings of disabled children and those with chronic illnesses and cancer, only a small number of studies have investigated the experiences and impact on psychosocial wellbeing of having a sibling with a life-limiting condition. The experiences of this group of siblings are likely to be different as a result of the inevitability of their sister’s/brother’s premature death, the complexity of their health needs and the constant risk of life-threatening events. Studies suggest that during childhood these siblings can experience a range of emotional and behavioural difficulties during childhood and have a lower quality of life compared to their peers.^7–12^ In addition, studies report that they may experience a lack of attention from parents which can lead to feelings of exclusion^8,12,13^ and that their social activities can be limited due to their own caregiving responsibilities.^10,12,13^ However, studies also identify positive aspects including an increased sense of maturity and enhanced qualities of compassion and empathy.^8,10,12,13^ During childhood siblings may also lack opportunities to express their feelings and needs^14^ and may lack information about their sister’s/brother’s condition and care.^15^

Little is currently known about the experiences and support needs of adult siblings of children/young people with a life-limiting condition (including those who are bereaved) and appropriate ways of providing support during adulthood. This is a significant omission given the increasing prevalence and life expectancy of children and young people with life-limiting conditions.^16^ This study was designed to address this gap in knowledge. Its aims were to explore adult siblings’ perspectives on their experience of having a sister/brother with a childhood life-limiting condition and to identify their perceived needs and preferences for support. As this population includes bereaved and non-bereaved siblings, both groups were included in the study to comprehensively understand their experiences and support needs.

## Research methods

A qualitative exploratory study design was adopted. Data were generated through semi-structured interviews and analysed using reflexive thematic analysis, underpinned by an interpretivist ontology/epistemology.^17^

### Population

The population were adult siblings who had a sister/brother with a childhood life-limiting condition.

### Setting

**Figure 1.**
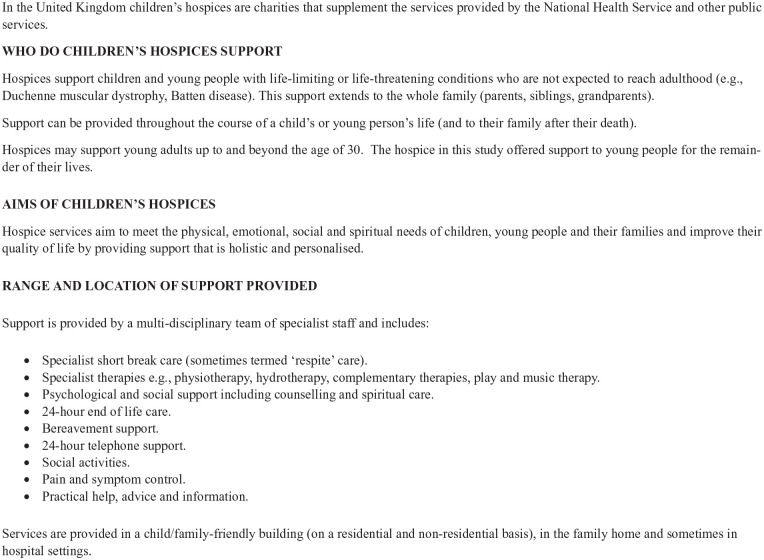
Characteristics of children’s hospices in the United Kingdom (UK).

Siblings were recruited via one children’s hospice in England. The characteristics of children’s hospices in the United Kingdom and the population they support is presented in Figure 1.

### Sampling

The sample was comprised of adult siblings meeting the study inclusion criteria (Table 1). No age restrictions were placed on the age of their sister/brother. In the first stage of the study adult siblings from families who had received support from the children’s hospice were invited to complete a questionnaire about their experiences and support needs (these findings are not reported in this paper). This incorporated a question for respondents to register their agreement to be approached about being interviewed and provide their contact details.

**Table 1.** Sample inclusion and exclusion criteria.

Inclusion criteria
Sibling of a child/young person with a life-limiting condition diagnosed during childhood (before their 18th birthday) who were:
• Aged 16 years and over (at the time of the study).
• Bereaved or non-bereaved.
• Where bereaved, the death of their sister/brother could have occurred at any age (including the neonatal period).
Exclusion criteria
Siblings
• Whose brothers/sisters had died within the previous 6 months.
• With a life-limiting condition themselves.
• Lacking mental capacity.
Families with current safeguarding concerns (where services were concerned about or investigating the safety of children or adults in the family)

### Recruitment

Thirty-three siblings agreed to be approached (58% of survey respondents (*n* = 57)) and were emailed a copy of the participant information sheet by the project researcher. They were contacted approximately one week later by telephone/email to discuss their willingness to be interviewed and arrange a convenient interview appointment. Twenty-two siblings subsequently consented to participate.

### Data collection

Due to the COVID-19 pandemic remote interviews were conducted (January–May 2021). Participants were offered the choice of telephone or video interviews, and all selected to be interviewed via telephone. Two topic guides were developed (one for bereaved siblings and one for non-bereaved siblings) and used to structure the interviews whilst also being flexible to allow for the pursuit of areas raised by the participants (Table 2). In the interviews siblings were invited to reflect on their experiences during childhood, adolescence and adulthood. All interviews were conducted by the project researcher (an experienced applied health researcher) who was unknown to the participants and had no connection to any organisation supporting the participants or their families. Interviews were securely audio-recorded with the participant’s permission.

**Table 2.** Interview topic guides.

Non-bereaved siblings	Bereaved siblings
*You and your sibling* • Can you tell me a little about you and your sibling (name of sibling)? • Can you tell me about your relationship with your sibling? ○ If/how relationship has changed ○ Future changes in relationship. • What was their life like? *Daily life and activities* • Tell me about your daily life and activities. • Tell me about any help you give to your parents and sibling. • Perceptions of impact on daily life? ○ Sleep/social activities/relationships with family/peers ○ How do they manage this? *School/work* • Can you tell me about school/work? • Impact on your education/employment? ○ Time off ○ Impact on school activities/grades/homework ○ Working hours ○ Reactions of peers/staff ○ How have they managed this? *Emotions* • Feelings about having a sibling with a life-limiting condition. ○ How do they manage this? • What is your biggest worry about as a sibling? • Can you describe positive/negative experiences of being a sibling? *Support needs and experiences* • Tell me about the support you have been offered/ accessed/received as an adult sibling. • Support received: ○ Accessibility ○ Usefulness/appropriateness ○ Positive experiences (what was valued) ○ Negative experiences (any difficulties) • Suggestions to improve support given to adult siblings. *Debrief*	*You and your sibling* • Can you tell me a little about you and your sibling (name of sibling)? • Can you tell me about the relationship you had with your sibling? • What was their life like? *Daily life and activities* • Perceptions of impact on daily life? ○ Sleep/social activities/relationships with family/peers ○ How did they manage this? • Support/help given to your family. • Daily life and activities now (any differences since their sibling’s death) *School/work* • Can you tell me about school/work? • Impact on education/employment? ○ Time off ○ Impact on school activities/grades/homework ○ Working hours ○ Reactions of peers/staff ○ How have they managed this? *Emotions* • Feelings about having a sibling with a life-limiting condition. ○ How do/did they manage this? • What was your biggest worry as a sibling? • How has the loss of your sibling with a life-limiting condition made you feel? ○ How do you manage this? • Did you feel prepared for the circumstances of your sibling’s death? • Can you describe positive/negative experiences of being a sibling? *Support needs and experiences* • Tell me about the support you have been offered/ accessed/received as an adult sibling. • Support received – before and after loss of sibling ○ Accessibility ○ Usefulness/appropriateness ○ Positive experiences (what was valued) ○ Negative experiences (any difficulties) • Suggestions to improve support given to adult siblings (before and after bereavement). *Debrief*

### Data analysis

The interview recordings were fully transcribed and imported into NVivo 12 for analysis using reflexive thematic analysis^17^ by both SK (a palliative care researcher) and SP (a mental health researcher). Following data familiarisation, initial codes were generated inductively and combined to develop potential themes. These potential themes were then reviewed and refined until a set of four themes were generated that SK and SP considered captured the most meaningful aspects of the data in relation to the research aims.^18^

### Ethical issues

The study was approved by University of Manchester Ethics Committee (2020-9244-16790). Informed consent was obtained prior to an interview and participants were given assurances of anonymity and confidentiality unless disclosures were made about a person’s safety. Pseudonyms are used in data extracts. Protocols were developed for managing safeguarding issues and any participant distress.

## Findings

Of the 33 siblings approached, 22 agreed to be interviewed (3 siblings declined to participate and 8siblings did not respond to emails/telephone messages). Their average age was 28.2 years (range 17–42 years) and 15 (68.2%) were bereaved siblings (Table 3). Living sisters/brothers ranged between 17 and 27 years old. The deaths of sisters/brothers had occurred between 3 and 24 years ago. The research findings are organised in relation to the four themes generated from the analysis.

**Table 3.** Participant characteristics (*n* = 22).

Characteristic	Frequency	Percentage (%)
Age (years)		
16–20	3	13.6
21–25	8	36.4
26–30	3	13.6
31–35	4	18.2
36–40	2	9.1
41–45	1	4.6
46–50	1	4.6
Mean: 28.2 years; range: 17–42 years		
Gender		
Female	13	59.1
Male	9	40.9
Ethnicity		
White	19	86.4
Asian/Asian British (Pakistani)	2	9.1
Mixed	1	4.5
Status		
Bereaved	15	68.2
Non-bereaved	7	31.8
Participant’s age in relation to brother/sister		
Older	16	72.7
Younger	6	27.3
Non-bereaved siblings – age of brother/sister (years) (*n* = 7)		
17–22	4	57.1
23–27	3	42.9
Mean: 21.9 years; range: 17–27 years		
Bereaved siblings – length of time since death of brother/sister (years) (*n* = 15)		
3–8	4	26.7
9–14	5	33.3
15–20	4	26.7
21–24	2	13.3
Mean: 13.2 years: range: 3–24 years		
Participant’s age at time of brother/sister’s death (years) (*n* = 15)		
5–11	5	22.7
12–18	4	18.2
19–25	4	18.2
26–32	0	0.0
33–39	2	9.1
Range: 5–39 years		
Age of brother/sister at the time of their death (years) (*n* = 15)		
2–8	5	22.7
9–15	5	22.7
16–22	2	9.1
23–29	2	9.1
30–36	1	4.6
Range: 2–36 years		
Condition/diagnosis of brother/sister (by ICD-10 category)		
Duchenne muscular dystrophy	4	18.2
Cerebral palsy	4	18.2
Cancer	3	13.6
Metabolic condition	3	13.6
Chromosomal abnormality	2	9.1
Congenital heart condition	2	9.1
Other	4	18.2

### Coming to understand the meaning of a life-limiting condition

Siblings described how family life had revolved around the needs of their sister/brother. Their sister’s/brother’s impairments made engaging in social activities outside the home challenging and over time this had become increasingly difficult as their condition worsened. In addition, siblings’ lives were often constrained and structured by their sister’s/brother’s treatment regimens. Despite this, when they were young children, they had viewed their lives as ‘normal’ because they had not experienced any alternative:

*When I was little, I sort of assumed that every other child my age had a sibling in a wheelchair. . . I thought that everybody else went to respites and spent time at hospital with their sibling. . . I just thought that was the norm because I didn’t know anything else. . . I thought every family had a disabled child*. Jess (age 19, bereaved 13 years)

However, over time siblings came to recognise that their family life was not the same as other families; social interactions raised their awareness of their sister’s/brother’s difference. In public settings siblings described people staring at their sister/brother and friends questioned them about their needs due to the presence of medical equipment in the home or because they attended a different school. As siblings became older, they described becoming aware that their sister/brother would have a shortened life-expectancy – an awareness that appeared to impact on their mental health:

*I didn’t have that worry as a child, I didn’t have that anxiety, I was quite carefree. . . but as I’ve grown up I have an understanding with what’s going on with X, his condition. That’s when my anxiety started to kick in, that really worried me and I think that’s when it all just started going downhill with my mental health.* Ayesha (age 17, non-bereaved)

*It affects every aspect of your life when you become old enough to realise that your sibling is different from other children and that their life will not develop as yours will. . . as you grow up and learn more about the condition that your sibling has the reality of it all hits home.* Kate (age 35, bereaved 18 years)

Participants described feelings of sadness at seeing their sister/brother’s health decline and anxiety over the uncertainty surrounding their condition and the timing of their death:

*I just worry about what’s going to happen because, obviously, they’ve just told us that X’s condition’s worsening and he’s deteriorating and there’s nothing that they can do. We just have to, like, wait.* Ayesha (age 17, non-bereaved)

*My biggest worry was that he was going to die. . . So it was just like a constant worry in my head, and it really did get in the way of everyday life. . . I suffered a lot of anxiety because I didn’t know when he would die and the thought of him suffering and dying gave me recurring nightmares. . . I always felt like I had to be around my brother 24/7 in case something happened to him. I still have this same mentality to this day. When I go somewhere I feel the urge to rush back home continuously to see my brother even though he’s not alive.* Ella (age 19, bereaved 3 years)

Even though siblings had developed an understanding that their sister/brother had a shortened life expectancy their death came as a shock to them and was experienced as unexpected and traumatic. For some participants the shock related to them having become used to their sister/brother being very ill and being admitted to hospital but then ‘pulling through’, as Mia explained:

*He was always okay, he always pulled through so when he actually did pass away when he was 17, even though we was expecting it all his life, it was still a shock because I was used to him pulling through all the time. . . even though you expect it, it’s still unexpected*. Mia (age 31, bereaved 13 years)

### Changing relationships and roles

Participants described how, over time, their relationship with their sister/brother had changed from a sibling relationship to a caring relationship. When they were children they recalled socialising together, however, the relationship became more defined by caring as their sister’s/brother’s condition deteriorated and their needs increased and as siblings became older and assumed responsibilities for their sister’s/brother’s care. Siblings with living sisters/brothers felt that their caring responsibilities were increasing as their parents were becoming less physically able to care for their adult child with a life-limiting condition. At the same time participants felt that these experiences had led them to becoming more caring, empathetic, mature and independent than their peers. For some, caring for their sister/brother had influenced their career/employment choices.

As participants grew-up they described providing practical support for their parents such as helping with household chores and caring for sisters/brothers (including those with a life-limiting condition) to give their parents a break or to allow them to go shopping, go to work or complete household chores. Emily reflected on how she had adopted the role of ‘helper’ in the family which she felt had enabled her to feel part of the family:

*I would like help out. I think that became my role as like the helper, that was my role in the family unit. I felt that helping was my way of being like part of it. . . I would help with bits round the house and stuff.* Emily (age 24, bereaved 17 years)

Sophie described how she had started caring for her parents following her sister’s death and their deteriorating mental health:

*I go round to my mum and dad’s every morning, cook them breakfast because my mum literally can’t walk now. . . they both have depression. But I go around every day and I do the cleaning and I go round at night-time and put the tea on*. Sophie (age 42, bereaved 6 years)

In adulthood siblings described providing emotional as well as practical support for parents:

*It’s emotional support, as well. So, my mum, I do a lot of that, because she’s a single parent she’s been through a lot and I think she just needs it because there are so many of us, and there’s X [*brother*], obviously. And I’ve been there a lot for her.* Ayesha (age 17, non-bereaved)

*My mother went into shock [after sibling’s death] and she developed mental illness. So it was putting her first. . . My mother’s mental health has suffered dramatically and more time is required to support her.* Amelia (age 46, bereaved 7 years)

Siblings felt that their sisters’/brothers’ caring needs had influenced their relationship with their parents and they perceived experiencing a lack of attention from their parents. This parental emotional and physical unavailability could persist following their sister’s/brother’s death and affect their relationships with parents in adulthood. As Alice and Emily explain, this experience made siblings feel that they and their needs had been marginalised within the family which had impacted on their mental health:

*The attention was all on X. I suppose I did feel quite lonely and upset and maybe even angry that all the attention was on him. . . I felt depressed for a long time because of my brother’s condition because I was never involved as much as he was. . . He is the centre of attention.* Alice (age 25, non-bereaved)

*From birth having to put somebody else’s needs before your own and you’re not the main focus and stuff like that, I think that affects a person.* Emily (age 24, bereaved 17 years)

However, siblings described how as adults they had developed an understanding of their parents’ actions in prioritising their sisters’/brothers’ needs:

*I did get a bit jealous that he needed their attention more but then I think as I got older I kind of understood that it wasn’t that they were giving him the attention because they wanted to, it was because they had to*. Eve (age 24, bereaved 3 years)

From participants’ accounts the sibling-parent relationship appeared to be defined by protection. Siblings were concerned about their parents’ emotional wellbeing so they protected them emotionally by not disclosing their own feelings about how their sister’s/brother’s condition or death affected them:

*They are going through grief, so they’re like debilitated by the grief. . . you don’t tell your parents that you feel like that too, do you, when you’re that age. . . my mum, she was so grief-stricken, I think like you just wondered if your problems were secondary to that*. Emily (age 24, bereaved 17 years)

*I don’t really speak to my parents about it* [anticipated death of brother] *because obviously they’re very sensitive subjects. . . I don’t want to put more worry on them. We all know it’s there. It’s like the elephant in the room, we all know it’s going to happen, so we kind of just put on a happy face and try and make the most of what time he has.* Leo (age 25, non-bereaved)

Protection also involved siblings trying to be ‘perfect’ and not create additional concerns or distress for their parents:

*I always wanted to be the perfect role model, so that people wouldn’t worry about me, or anything like that, and they could just focus on my brother. And I think that that kind of desire to be perfect is really unachievable, especially in a young child. . . those kind of expectations to kind of grow up quicker, be a role model, be a good person, they kind of forced me to grow up at an early age.* Ella (age 19, bereaved 3 years)

*I worried a lot about making the right choices all the time, because I didn’t want to put any more worry or any more burden on my mum and dad. Like I couldn’t have, if I’d upset them for whatever reason, I played up, in the back of my mind, I was always thinking, no, I can’t do that to Mum and Dad. . . I was always thinking, well, no, my mum and dad have so much to deal with already.* Kate (age 35, bereaved 18 years)

### Developing an understanding of emotions and support needs

Participants described how they were only able as adults to retrospectively understand the impact that having a sister/brother with a life-limiting condition had had on their mental health. Siblings reflected on the difficulties they had experienced when they were younger in understanding their feelings:

*I probably didn’t recognise it. Because obviously, when you’re starting your teenage years, everything seems so important, all the time, every little thing. So I feel like I remember I would get really emotional about things. I don’t think I was an anxious pre-teen, but I was definitely quite emotional. And I recognise now that, that was something to do with X.* Jess (age 19, bereaved 13 years)

*I realised* [when accessing university counselling] *that I also had more emotional trauma through X’s hospitalisations and the fact that he needed more attention from my parents than I did. However, it was never something I realised while growing up.* Eve (age 24, bereaved 3 years)

Bereaved siblings described being unprepared for or not understanding the emotions they experienced after the death of their sister/brother.

*I definitely wasn’t prepared for the after bit, of grief and stuff, I don’t think I understood what it was, until I went through it. And even when I was going through it, I didn’t really realise what it was.* Jess (age 19, bereaved 13 years)

*I carried that [*guilt*] around with me, why am I thinking like this? Why am I wishing death upon my brother who I love? It doesn’t make any sense. . . why was I was thinking and feeling the way I sort of did.* Harry (age 34, bereaved 15 years)

In retrospect siblings realised that they would have benefited from emotional support earlier in their lives. Some felt that not receiving emotional support or psychotherapy at the time, either because it was not offered or they had declined it, may have led to longer term mental health problems or unresolved emotions.

*In hindsight probably looking back at my younger self, I probably would have suggested some form of bereavement counselling to deal with that. Because I don’t think I ever really did. . . maybe bereavement counselling at that time would have helped me deal with X, and maybe I would have had a different outcome maybe. I would have learnt to deal with things in a different way, and process them, and deal with my emotions better*. . . *I think a lot of people don’t realise how much their past experiences affect them in adulthood.* Olivia (age 26, bereaved)

*I had no outlet to talk to anyone at that point about anything like that, so I sort of carried that around with me, and that’s something that I wish I could have addressed at the time. . . I would have benefitted from talking to someone when I was a teenager.* Harry (age 34, bereaved)

While some participants described being able to talk about their feelings to others, many found this difficult. As discussed earlier, siblings were reluctant to disclose their feelings to their parents. Ella explained how siblings could also find it difficult to talk to friends about their feelings and experiences because they felt the reality of their lives would not be understood:

*I oftentimes felt very alienated from my peers. . . they weren’t going through the same experience as me, and I didn’t know anyone who was going through the same experiences as me, so I felt like I had no one to talk to.* Ella (age 19, bereaved)

Ben and Ella described ‘bottling up’ their feelings and putting a ‘mask on’. Ultimately both experienced severe depression with Ben being admitted to a mental health hospital.

*I used to bottle it up until, probably, I’d say about the last two years. So, just about a year ago, I went through a really bad time, ended up in hospital and then, after that, I kind of, opened up to my family.* Ben (age 23, non-bereaved)

*I had to kind of put on this new, I guess, mask, every day, to try and convince people that everything was fine, when in reality, it wasn’t. Especially at school, I had to basically pretend to everyone, especially my peers, that my life was fine when it wasn’t.* Ella. (age 19, bereaved)

Siblings recalled not taking up offers of emotional support from services because they did not feel able to talk about their feelings or did not recognise the benefits of this in adolescence.

*I do remember one member of staff saying if you ever want to speak to anyone, you can, and I kind of brushed it off because again I didn’t want to speak at that time. . . I think when you’re a bit of a moody teenager you don’t want to speak about things you’re going through. I think you bottle it up.* Mia (age 31, bereaved)

*At the time, I think I pushed an awful lot under the rug. You’re 17, you’re not really thinking of the future or the bigger picture, you’re just thinking of the here-and-now, and oh god, if I start talking about something now, it’s going to make me cry and make me feel like crap, so I’m not going to do that, whereas, now, I’d think, well, have a good cry, and you’ll feel better tomorrow. At that age, I believe that you don’t really know what’s good for you. . . I think as you get older you just realise that maybe I didn’t deal with it.* Kate (age 35, bereaved)

The timing of the offer of bereavement counselling also appeared to be important as some participants reported declining it due to it being offered too soon after the death.

### Experiencing continuing distress

Bereaved siblings and those with living sisters/brothers described current feelings of anxiety and depression, which had persisted from childhood into adulthood, and for some had necessitated treatment including hospitalisation. Mental health problems could worsen as they got older, which some participants associated with life transitions such as starting college/university. Bereaved siblings related their current feelings of depression and anxiety to the death of their sister/brother which could lead to suicidal thoughts and self-harm:

*After my brother passed, I fell into a deep depression and I developed severe anxiety. I would get nightmares about my brother dying and these images about him suffering would pop up in my head all the time regardless of where I was. I still get the same nightmares to this day. During college it was hard for me to adjust to not only a new school but also a world without my brother. It was hard returning to ‘normality’. It got so hard alongside my depression and anxiety that I contemplated suicide many times. I just wanted to see my brother and thought that by dying not only would I get to see him but I would also be free from the pain that his passing caused me.* Ella (age 19, bereaved 3 years)

*I feel that I have been mainly impacted since X [*brother*] died. I had panic attacks prior to X’s death, but they were infrequent. Since his death I have had social anxiety, more frequent panic attacks, thoughts of self-harm, depressive episodes and disassociation. I struggled continuing my final year studies at university. Eve* (age 24, bereaved 3 years)

Although some siblings had accessed adult mental health services or received psychotherapy as adults (e.g. at university), participants felt that there was limited emotional support available for adult siblings unless they experienced a mental health crisis. Despite having continuing support needs, participants described how support stopped when they reached adolescence. Support often continued to be available to their parents and younger siblings which made them feel excluded. Participants described the importance of hospices maintaining a connection with siblings as they moved into adolescence and adulthood, contacting them directly (not via parents) periodically to assess their support needs and offer support.

Regarding adult sibling support needs, participants identified the need for psychotherapy and peer support. Psychotherapy was seen as being important in helping siblings understand their feelings and experiences both in the past and currently. Jess highlighted how she did not know how she should feel thirteen years after her brother died.

*I don’t really know much about, like, how, like, you’re supposed to feel after, like, 10, 15 years like, and obviously people say it gets easier, but I feel like counselling could really help in, like, oh it’s okay if it doesn’t seem to get easier.* Jess (age 19, bereaved 13 years)

Siblings emphasised the importance of therapists understanding the context of their lives; either in terms of knowledge about life-limiting conditions or having known their sister/brother and family. This meant that children’s hospices were felt to be best placed to provide psychotherapy.

*You’ve always got that thing in the back of your mind of, well, do they have any knowledge of what it’s like to grow up with a sibling that had special needs, but I suppose with* [a children’s hospice*], you would automatically just feel more comfortable, and a bit more at ease, because you’ve already got that feeling of, oh, they understand. . .They kind of know what it’s like, because they’re in the situation all the time.* Kate (age 35, bereaved 18 years)

Siblings described how online or face-to-face opportunities to meet with other siblings with a sister/brother with a life-limiting condition would enable them to talk about their feelings with a group of people who shared the same experiences and understood their situation. As Olivia explained peer support would help siblings feel less alone:

*It would be a great thing for the siblings to be able to say what they want to people who would understand. It’s not a lot of people have disabled siblings, and sometimes you can feel quite alone. I don’t know anyone with a disabled sibling. . . something that I could go to if I feel sad about my sister, or thinking about her, and I could put my thoughts out there, and there’d be people who understood.* Olivia (age 26, bereaved 18 years)

Ella described how peer support would also provide an opportunity to share coping strategies as well as feelings.

*It would be with people who are the same age, or similar age, and they’ve all probably gone through the same thing, and they could sit and talk through things. . . maybe, discussing healthy coping strategies, or something like that, just to show that you’re not alone in this, and it’s okay to feel like that, and you shouldn’t be ashamed to feel the way that you do. . . it would be nice to feel like there is a community of people out there who have been through the same thing as you and are there to help.* Ella (age 19, bereaved 3 years)

## Discussion

### Main study findings

Interviews with adult siblings have illuminated how the experience of having a sister/brother with a life-limiting condition changes over time as they come to understand the meaning of a life-limiting condition and as their roles and relationships with parents and sisters/brothers evolve. The study has revealed the enduring impact of these experiences on siblings’ mental health and their unmet support needs in adulthood. Siblings described the importance of support continuing throughout adolescence and into adulthood with a focus on the provision of psychotherapy and peer support. Children’s hospices were seen as being best placed to provide this support because of their understanding of the context of siblings’ lives.

### What this study adds?

Previous studies have explored siblings’ experiences of having a sister/brother with a life-limiting condition during childhood.^7–13^ This study contributes to knowledge by examining their experiences and support needs in adulthood. In addition, the study has provided a temporal perspective on these experiences, illuminating how their experiences, understandings, roles and relationships change over time and providing insight into their support needs during earlier stages of their lives.

This study adds to knowledge by revealing the profound and enduring impact of having a sister/brother with a life-limiting condition on the mental health of bereaved and non-bereaved siblings. It has highlighted how during childhood and adolescence they experienced difficulties understanding their emotions and recognising their need for emotional support. This coupled with both a lack of opportunity and a reluctance to express their feelings appears to have led to a continuation, if not worsening, of mental health problems in adulthood. There is evidence that the death of a sibling in childhood/adolescence is associated with an increased risk of mental health problems in adulthood and an increased mortality rate possibly due to high levels of loss-related stress and lack of support.^19,20^ In this study, it was notable that adult siblings with living sisters/brothers also reported experiencing mental health problems. Further research is needed on the prevalence of mental health problems in this group of adult siblings. A previous study has similarly reported that siblings lack opportunities to express their feelings in childhood^14^; the study reported in this paper has discovered how this may then lead to siblings experiencing difficulties in understanding their feelings, rejecting support and enduring mental health problems. This highlights the importance of palliative care services providing, and encouraging the uptake of, emotional support for siblings during childhood/adolescence to help them understand, recognise and express their emotions to prevent or ameliorate long-term mental health problems.

In this study protection and marginalisation appeared to have characterised sibling-parent relationships during childhood/adolescence. Previous studies have reported that during childhood siblings’ needs may be overlooked by parents.^8,12,13,21–23^ This study adds to knowledge by revealing how this can go onto shape parent-child relationships in adulthood that are also transformed by siblings’ increasing role in caring for their parents. This is of concern as the quality of the relationship between siblings and parents is a significant predictor of sibling mental health.^24^ The tendency of siblings to ‘silence’ their own emotions to protect their parents may mean parents are unaware of or overlook their emotional needs creating barriers to siblings receiving appropriate support. This may be exacerbated if parents believe, that by not discussing the impact of having a sister/brother with a life-limiting condition, they are protecting siblings from distress. Poor communication within the family and lack of social support are leading risk factors for unresolved emotional difficulties in siblings.^23^ Palliative care services need to promote and facilitate open parent-child communications to help increase siblings’ emotional sharing and their understanding of their sisters’/brothers’ life-limiting condition as well as raising parental awareness of their needs. Developing family-based interventions may be useful in improving parent ***-*** sibling communications and relationships and preventing later sibling mental health problems and is an area that needs further research.^25–27^

As siblings’ needs can be overlooked by parents and services, it is important that their individual needs are assessed as part of a holistic family assessment. In doing this palliative care services could take a person-centred, support needs approach (developed in adult palliative care) where individuals are facilitated to identify, express and prioritise their unmet support needs.^28^ A comprehensive psycho-social assessment, using recognised age-appropriate tools (alongside professional judgement) may help identify those at risk of mental health problems and facilitate timely support.

This is the first study to investigate the support needs of adult siblings with a sister/brother with a childhood life-limiting condition. It adds to knowledge by identifying their need for psychotherapy provided by those with experience and understanding of the context of their lives. From participants’ accounts it appears this may need to include psychotherapy for traumatic and complicated grief. This may mean that palliative care services need to develop or expand psychotherapy services. Further research is needed regarding effective support interventions for bereaved adult siblings in general^29^ and in relation to those with specific bereavement experiences such as having a sister/brother with a life-limiting condition.^30^ Another area identified by participants was the provision of peer support where siblings could share their feelings, experiences and coping strategies with a group who fully understands their situation and help reduce their sense of isolation. Peer support can take many forms but there is some evidence that the most promising types are: face to face groups run by trained peers; one-to-one peer support offered face to face or by telephone; and online forums.^31^ However, research is needed to investigate which peer support model would be effective and acceptable for adult siblings with a sister/brother with a life-limiting condition.

### Strengths and limitations

This unique study’s in-depth nature has illuminated the experiences and support needs of a group of siblings previously overlooked by research as well as providing insight into their experiences/needs during childhood/adolescence. A diverse sample was recruited although as they were recruited from a single hospice their perspectives on support may reflect a particular setting and service. They are also a self-selected sample which means that their characteristics and perspectives may be different to the wider population of adult siblings. Furthermore, conducting the study during the Covid-19 pandemic will have influenced the data generated; firstly, as a result of remote interviewing and secondly it may have influenced their accounts as lockdowns had led to some siblings providing additional support to their sisters/brothers and parents.
